# Case of Tetanus Treated with Calabar Bean

**Published:** 1877-01

**Authors:** 


CASE OF TETANUS TREATED WITH CALABAR BEAN.—H. W., aged forty-seven, a gardener, received a wound in the web between the thumb and index finger, from a branch of a laurel bush that had been cut off obliquely. The wound was about half an inch deep. It was carefully cleansed and kept open for a fortnight. Sixteen days after the injury symptoms of tetanus set in, and five days later, on June 16, 1875, he was admitted into St. George's Hospital, under the care of Dr. Dickinson and Mr. Pollock. At that time the symptoms of tetanus were well marked. He was in a condition of opisthotnos, and spasmodic contractions of the muscles of the neck and face, lasting about five seconds, occurred ten or twelve times a day. He was at first put on twelve grains each of chloral hydrate and bromide of potassium, with temporary improvement. On June 19th, however, he was much worse, the spasms recurring five or six times a minute. One-eighth of a grain of extract of Calabar bean was injected subcutaneously every hour, from 11:15 P.M. until 7:15 A.M., on the 20th. After this the spasms occurred at intervals of about two minutes, and were less severe. He felt easier, and slept some. On the 22d the injections were given everp hour and a half, alternately, with a pill containing one-sixth of a grain of the extract. After 10 P.M. the pills and injections were repeated every two hours. On the 24th, the improvement still continuing, the injection was increased to one-fourth of a grain. On the 25th the treatment was discontinued for five hours, but as the spasms became more frequent, it was resumed as before. On the 26th the spasms did not occur, except when the fauces were tickled, and the injection was given only every four hours. On July 1st the injection was ordered every third hour during the day, and a pill every third hour during the night. On July 3d there was a slight return of spasms. Two-thirds of a grain of the extract was given subcutaneously every three hours during the day, and as a pill at night. On the 4th one grain of the extract as a pill was ordered every three hours during the night; the injections as before. On the 5th the injection was increased to one grain, and the pill to one grain and a half; and on the 9th the injection was increased to one grain and a half, and the pill to two grains, given as before. The spasms now stopped, but the patient continued to have some pains in the back for three weeks, and his legs were still weak and stiff on August 18th, when he was sent to the convalescent home. He was seen some months afterward in good health.—*Western Lancet.*

